# Bayesian phylogenetic inference of HIV latent lineage ages using serial sequences

**DOI:** 10.1098/rsif.2023.0022

**Published:** 2023-04-19

**Authors:** Anna A. Nagel, Bruce Rannala

**Affiliations:** Department of Evolution and Ecology, University of California, Davis, CA, USA

**Keywords:** HIV, latency, Bayesian phylogenetic inference

## Abstract

HIV evolves rapidly within individuals, allowing phylogenetic studies to infer histories of viral lineages on short time scales. Latent HIV sequences are an exception to this rapid evolution, as their transcriptional inactivity leads to negligible mutation rates compared with non-latent HIV lineages. This difference in mutation rates generates potential information about the times at which sequences entered the latent reservoir, providing insight into the dynamics of the latent reservoir. A Bayesian phylogenetic method is developed to infer integration times of latent HIV sequences. The method uses informative priors to incorporate biologically sensible bounds on inferences (such as requiring sequences to become latent before being sampled) that many existing methods lack. A new simulation method is also developed, based on widely used epidemiological models of within-host viral dynamics, and applied to evaluate the new method—showing that point estimates and credible intervals are often more accurate than existing methods. Accurate estimates of latent integration dates are crucial in relating integration times to key events during HIV infection, such as treatment initiation. The method is applied to publicly available sequence data from four HIV patients, providing new insights regarding the temporal pattern of latent integration.

## 1. Introduction

A major obstacle to the development of a cure for HIV has been the presence of latently infected cells. HIV is a retrovirus that integrates its genome into the host genome. During latent infection, the integrated provirus is in a reversible state of transcriptional inactivity. Latently infected cells are not targeted by current treatment methods, namely antiretroviral therapy (ART). Consequently, treatment must be continued for life or reactivation of latent cells will lead to a rapid rebound in viral load and disease progression [1]. A detailed understanding of the dynamic processes of seeding, reseeding and decay of the latent reservoir through the inference of latent integration dates for individual proviruses will allow researchers to better understand the nature of the reservoir as they work towards a cure for HIV.

HIV infects immune cells, specifically CD4+ cells, such as helper T cells and macrophages. Most infected cells die quickly [2,3]. In contrast, memory T cells have a long half-life of 4.4 years and can thus establish a latent reservoir for HIV [4]. Memory T cells may be infected directly or an activated T cell may revert back to a quiescent state [5]. Latently infected memory T cells can be activated by antigens, leading to the activation of the HIV provirus [6]. Effective ART prevents infections of new host cells but does not prevent previously infected cells from producing virions. HIV can, therefore, persist hidden in memory cells for decades, even with effective ART [4].

The latent reservoir is initially formed within days of infection and continues to be reseeded over time [7–9]. However, the extent to which the composition of the reservoir changes over time is unclear. There is strong evidence that there is not ongoing cycles of viral replication during ART [10–12], so it is very unlikely the HIV reservoir is replenished from ongoing replication during ART. Some studies concluded that the latent reservoir that exists during ART is mostly seeded shortly before treatment initiation [13–15], while others have concluded that the reservoir is continuously seeded until treatment initiation [16]. However, some of these results are difficult to interpret as a variety of mechanisms could account for these patterns. The timing of the formation of the latent reservoir is ultimately an empirical question that can be studied in multiple ways. Experimental techniques, such as the use of quantitative viral outgrowth assays (QVOAs), allow researchers to obtain sequence data from individual cells known to be latent. In addition to further experimental work, reconstructing the ages of latent lineages can in principle be done by analysing the patterns of variation observed among sampled sequences and applying phylogenetic methods designed to estimate sequence divergence times with serial sequence samples [14–19]. The focus of this paper will be the development of new statistical and computational methods to accurately date the integration times of sampled latent sequences.

A variety of heuristic methods have been developed to estimate integration times using a combination of RNA sequences from serially sampled actively replicating sequences and RNA or DNA from putative latent sequences. All methods rely on a fixed estimate of the gene tree topology for the HIV sequences and some require branch lengths. Jones *et al.* developed a distance method that used linear regression (LR) to estimate the mutation rate from root-to-tip distances and sampling dates for non-latent sequences. This mutation rate is then used to estimate the latent integration dates [16]. This method relies on a molecular clock, and is not used if the clock is rejected. Jones & Joy [18] developed a related method, estimating mutation rate in the same way but estimated internal node ages using a maximum likelihood (ML) approach using a specified mutation rate. To *et al.* [20] developed a distance method using a least squares (LS) approach to estimate mutation rates and date internal nodes and tips with unknown ages. Their method requires the sequence length for estimating confidence intervals, but not the alignment. It was designed for extremely large phylogenies, but is applicable to HIV latency datasets as well. Abrahams *et al.* used multiple heuristic methods to date latent sequences. In one method, the distance from the closest sequence to the latent sequence, *d*, is determined, and the age of the latent sequence is assigned based on the sample time of the majority of sequences within 2*d* of the latent sequence [14]. A similar method traverses the tree from the latent sequence towards the root of the tree until a node with 90% bootstrap support is found with at least one pre-treatment sequence. Then a latency time is assigned based on the most common sampling time of the pre-treatment sequences descendant from the well supported node [14]. The two methods used by Abrahams *et al.* may be very sensitive to the number of sequences sampled and the sampling times. Simulation studies suggest that LS may out-perform all of these methods [18,20]. An alternative to these existing methods could be developed based on established parametric phylogenetic models that use tip dating for estimating and calibrating phylogenies of viral data, and are potentially more accurate [21,22].

It has been difficult to evaluate the statistical performance of current methods for inferring integration times of latent HIV since existing simulation methods are biologically unrealistic. During the acute phase of infection, viral load grows exponentially shortly after infection, peaking within several weeks [23]. Then the viral load falls one to two orders of magnitude before reaching a quasi-steady state. During this chronic phase of infection, the viral load remains relatively unchanged or rises only slowly until the onset of AIDS. By contrast, simulation methods that have been used to evaluate methods for dating integration events largely ignore the underlying population dynamics of HIV. Some assume a constant rate birth–death process while others use a compartmental model with logistic growth [16,18]. Epidemiologists use more complex models, typically ordinary differential equations (ODEs), to describe HIV viral dynamics [24–26]. These models produce population trajectories that more closely match empirical observations, especially during acute infection, but the models have yet to be used in simulations to generate within-host HIV sequence data. The time period of acute infection is known to be important in establishing the latent reservoir [7], and this peak dynamic should be incorporated into simulation methods used to test inference methods aimed at estimating latency times.

We propose a full likelihood Bayesian inference method to infer the latent integration date of HIV sequences, conditional on the phylogenetic tree topology. The method assumes it is known *a priori* which sequences are derived from latent proviruses and which are from non-latent viruses. This is possible when sequencing RNA from untreated patients and using QVOAs which stimulate the production of virus from latently infected cells. Additionally, we develop a simulation method based on existing viral dynamic models of HIV to test the performance of the inference method. The simulation model is parameterized using estimates from empirical datasets that produce realistic viral population dynamics (see material and methods) [27].

## 2. Model

A new program, HIVtree, was developed by modifying an existing program, MCMCtree, to infer latent integration dates [21]. MCMCtree is a Bayesian phylogenetic inference program which estimates a time calibrated tree using viral sequences with serial samples given a fixed tree topology. It uses Markov chain Monte Carlo (MCMC) to estimate the model parameters. HIVtree incorporates additional parameters, the latent integration times, into the model. The program also estimates the originally defined parameters in MCMCtree, including substitution model parameters, substitution rate and the internal node ages.

HIVtree assumes *a priori* that certain sequences are known to be latent while others are known not to be. Every sequence must also have a known sample date. In addition, every latent sequence has an unknown latent integration date. The youngest possible latent integration date is the sample time, and internal nodes cannot be latent. There is an optional bound on the oldest possible latent integration time, which could correspond to the oldest possible infection time. The model assumes that latent lineages have a mutation rate of zero, and all other lineages follow strict molecular clock. For calculating the likelihood, the latency time is treated as if it were the sample date for a non-latent lineage. This acts to reduce the tip age to be the time the sequence became latent (electronic supplementary material, figure S4).

### 2.1. Markov chain Monte Carlo

HIVtree adds an additional step to the MCMC to estimate the latent times. In MCMCtree, proposals to non-root internal node ages are bounded above by the age of the parent node and below by the age of the oldest daughter node. A new time for each internal node is proposed within these bounds, the acceptance ratio is calculated and the move is either accepted or rejected [21]. In HIVtree, in addition to bounds on nodes, latent times are bounded above by the age of the parent node and below by the sample time. This ensures that the sequence becomes latent before it is sampled and that internal nodes cannot be latent. If the optional bound on latent integration times is used, the younger of the parent node age and the bound is used as the bound. Similar to MCMCtree, for each latent time, a move is proposed within these bounds, the acceptance ratio is calculated and the move is either accepted or rejected (electronic supplementary material, figure S4). Other than the difference in bounds, the proposal moves for the internal nodes and the latency times are identical. For the mixing step, the latency time is treated as equivalent to the sample date. The mixing step was not modified from MCMCtree [21]. The implementation was validated with Bayesian simulations (electronic supplementary material, section S7).

### 2.2. Prior on distribution of times

Two new root age priors were implemented in HIVtree. HIVtree and MCMCtree both require the user to specify the priors in backward time. The time of the last sample is considered to be time zero, and earlier times are positive. The programs also require a specification of a time unit transformation. For example, consider HIV data with the sample times specified in days. A time unit of 1000 days means that 0.365 is equivalent to a year in the prior specification. A shifted gamma prior, Γ(α,β), is implemented as the root age prior. The distribution is shifted by adding the earliest sample time to the variable. This ensures there is no density for a root age younger than the sample ages. The gamma distribution parameters must also be chosen with the time unit transformation going backward in time. An option for a more informative prior is a uniform prior with narrow hard bounds (zero tail probability), *U* (*a*, *b*). There is no explicit prior on the internal nodes ages which is equivalent to a uniform prior on the possible node ages given the constraints from the sampling dates and the root age. This is in contrast to MCMCtree, which uses a birth–death sequential sampling prior [21]. Since the sampling prior is not explicit and the rank order of the nodes and the constraints jointly determine the prior, the MCMC must be run without data in order to recover the prior for the internal nodes, latency times and root age. The distribution of the root age when the MCMC is run without data will not be equivalent to the user specified prior (electronic supplementary material, figure S5). This results from the lack of an explicit prior on the internal nodes and latency times and from not explicitly conditioning on the tip ages (electronic supplementary material, section S13). This effect is similar to constraints imposed by fossil calibrations [28]. The mean root age will be older than the expectation of the prior distribution. The parameters of the gamma distribution can be modified to achieve a desired mean and variance for the root age. Using a uniform prior with a wide interval is discouraged due to this effect (an induced prior age of the root that is very old).

### 2.3. Combining inferences across genes

HIVtree only allows single locus inferences and assumes no recombination within a locus. However, recombination is common in HIV, meaning the whole genome cannot be analysed assuming a single gene tree topology. However, the entire HIV genome is incorporated in the host cell genome at the same time, meaning different regions of genome share the same latent integration times. Let *X* = {*x*_*i*_} be sequence data for *n* loci, where *x*_*i*_ are sequence data at locus *i*. Let *T* be a latency time that is shared across loci. The remaining parameters of the gene tree may be different due to recombination between loci. The posterior density of *T* is

$$f\;(T|X)=\frac{P(X|T)f\;(T)}{\int P(X|T)f\;(T)\;dT}.$$

If we ignore the correlation between gene trees due to limited recombination and treat the loci as independent, as is generally done in phylogenetics, the posterior density can be written as

$$f\;(T|X)=\frac{\prod_{i=1}^{n}P(x_{i}|T)f\;(T)}{C_{A}},$$

where *C*_*A*_ is the marginal probability of the data (which is a constant),

$$C_{A}=\int \prod_{i=1}^{n}P(x_{i}|T)f\;(T)\;dT.$$

We want to calculate the posterior probability of *T* for each locus separately using MCMC and subsequently combine them to obtain a posterior density for all the loci. To do this, we formulate the above equation as a product of the marginal posterior of *T* for each locus,

2.1 $$f\;(T|X)=\prod_{i=1}^{n}\left[\frac{\;f\;(T|x_{i})}{f_{i}(T)}\right]\times f\;(T)\times \frac{\prod_{i=1}^{n}C_{i}}{C_{A}},$$

where *f*_*i*_(*T*) is the prior on *T* for the *i*th locus and *f*(*T*) is the desired prior for the combined posterior. The last term is a proportionality constant that insures the posterior density integrates to 1. *C*_*i*_ is the marginal probability of the data for an individual gene,

$$C_{i}=\int P(x_{i}|T)f\;(T)\;dT.$$

A simple example illustrating this general approach to combine posteriors using a normal distribution is provided in electronic supplementary material, section S9.

In our analyses, *n* independent MCMC analyses are run (with and without using the likelihood) and kernel density estimation is used to estimate *f*(*T*|*X*_*i*_) and *f*_*i*_(*T*), respectively, for *i* = 1, …, *n*. The estimated kernel functions are then used to evaluate equation (2.1) up to an unspecified proportionality constant (see electronic supplementary material). Simulations were used to evaluate the performance of this approach to combine posteriors.

This method may be used on regions of the genome that are not complete genes. For simplicity, the term gene tree will be used to describe a phylogeny inferred using data from any region of the genome, but genomic region will be used rather than gene to describe a part of the genome that may not produce a complete functional product.

## 3. Results

### 3.1. Simulation analysis

Here, we compare the statistical performance of HIVtree and several other existing methods when analysing simulated datasets with known latency times.

#### 3.1.1. Comparisons on a fixed tree topology

**Figure 1.**
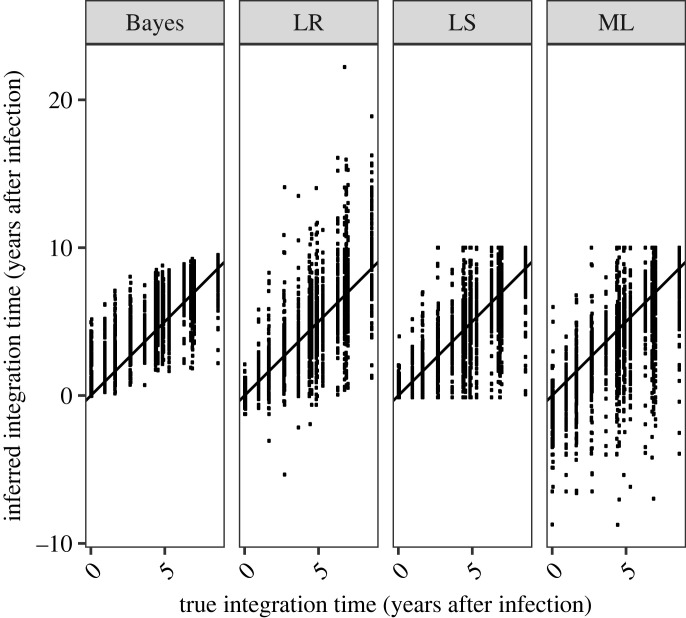
For all 30 alignments simulated for *C1V2* on a fixed tree, the inferred integration dates are shown for each method. If the methods performed perfectly, all points would fall on the line, which is has an intercept of 0 and slope of 1. The units are years after infection.

HIVtree was compared with three existing methods, LS dating [20], LR [16] and pseudo-ML [18] using simulated datasets. The effect of variation among the independently simulated sequences on point estimates of latent tip ages can be seen by comparing the estimates for a given latent tip in a fixed tree. Even with *C1V2*, the most informative genomic region simulated, there is considerable variation in the estimated latency time for a given latent tip (figure 1). The variation is even larger for the other genomic regions (electronic supplementary material, figure S13). The estimated times for a single latent tip sometimes differs from the true value by a decade or more for both the LR and ML methods. The LS method has fewer extreme estimates, which are prevented by bounds on the integration times. LS allows for upper and lower bounds for each individual latent sequence while ML has the same upper bound on all latent sequences, which is the last sample time. The LR has no bounds on the inferred integration time, potentially allowing the latent sequences to be formed either after the sequence was sampled or before an individual was infected. Both outcomes are highly unlikely.

#### 3.1.2. Combined inferences across genes

**Figure 2.**
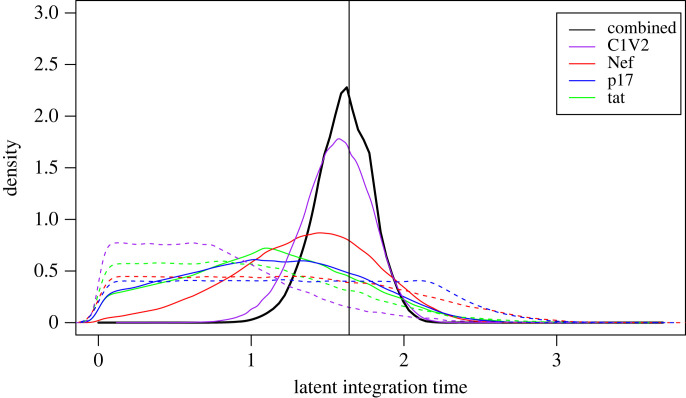
Joint posterior density for a single latency time across all simulated regions. Each solid coloured line shows the marginal posterior density for a single latency time for different genomic regions. The dashed coloured lines show the marginal prior densities, which result from running the MCMC without data. The solid black line shows the estimate with the genomic regions combined. The vertical line is the true latent integration time. The MCMC was run for 500 000 iterations, sampling every other iteration. This results in smoother curves than the shorter MCMC runs used in the larger analysis of simulated data, but results are very similar.

The posterior distribution for each latent time is inferred separately for each genomic region when using HIVtree. When the marginal densities are combined across the regions, the posterior densities become narrower and closer to the true value (figure 2). The other methods presented here do not allow such information sharing.

#### 3.1.3. Summary of method performance

**Figure 3.**
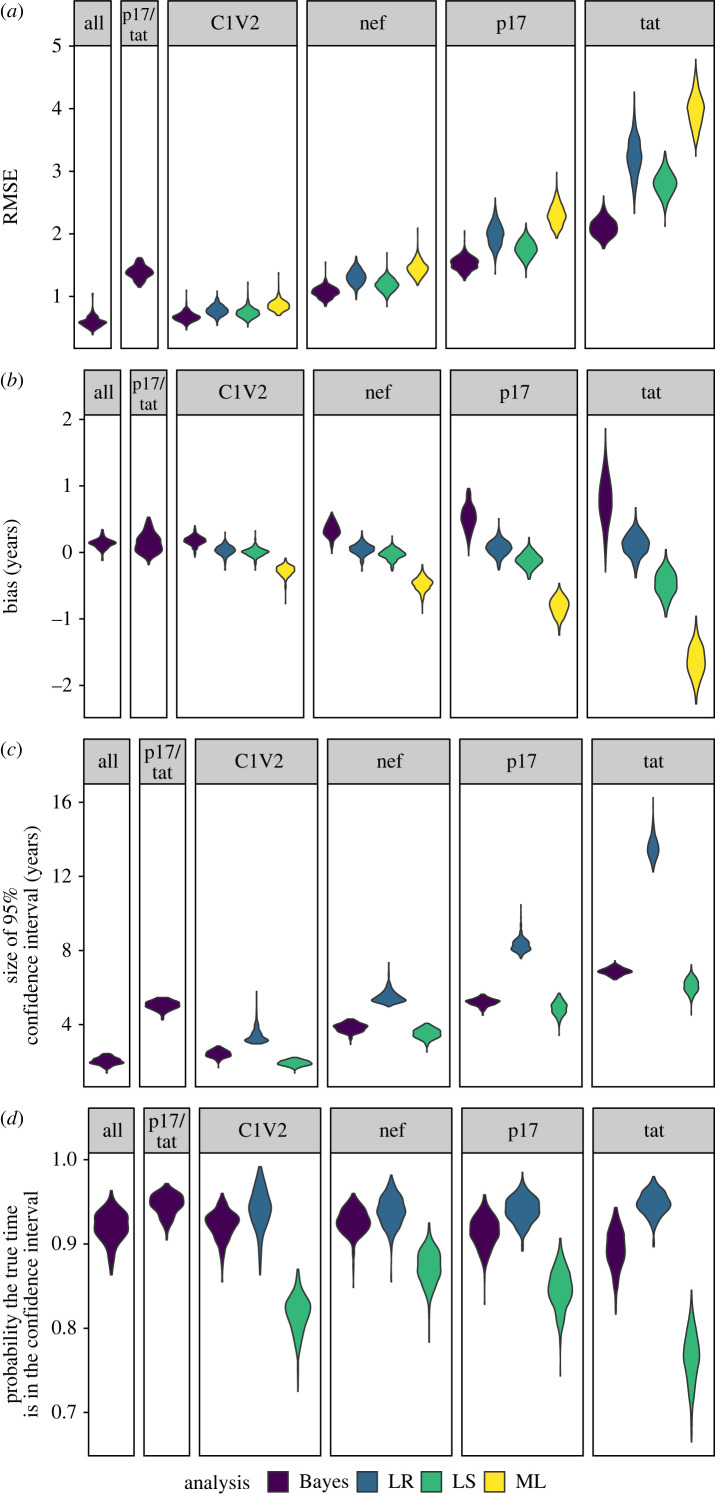
For each of fixed tree topologies, the root mean square error (RMSE), bias and size of the 95% confidence/credibility interval was averaged across all 900 latent times for each genomic region analysis combination. Each violin plot is made using 300 data points, corresponding to the average from each of the 300 fixed tree topologies. For the Bayesian combined analysis of either all of the genomic regions or only *p17/tat*, only a third of the fixed tree topologies were analysed.

Root mean square error (RMSE) is a useful measure of method performance that includes both bias and variance and is directly comparable across methods. RMSE is lowest for *C1V2* and highest for *tat* for all analyses (figure 3*a*). For *C1V2*, the average RMSE among methods, from lowest to highest, is Bayesian (0.67), LS (0.74), LR (0.77) and ML (0.86). All the methods are the least biased for *C1V2* and most biased for *tat* (figure 3*b*). The average bias for the ML and LS methods are more negative for the shorter, slower evolving genomic regions (−1.64 and −0.47 years, respectively, for *tat*), while the Bayesian and LR method have a positive bias on average (0.78 and 0.12 years, respectively, for *tat*). The trend for the mean square error (MSE) is similar to the trend for RMSE (electronic supplementary material, figure S36).

The probability that the true value falls in the 95% confidence interval (or 95% highest posterior density interval for Bayesian analysis) was also considered (figure 3*d*). The Bayesian method has comparable average coverage probabilities for *C1V2* and *nef* of 92% and 93%, respectively, with the lowest coverage probability for *tat* (89%). The average size of the 95% credible set for the longest and shortest sequences, *C1V2* and *tat*, are 2.4 years and 6.9 years, respectively. LR has the highest coverage, with a coverage probability of 94% for *C1V2* and 95% for *tat*. However, LR has very large confidence intervals (figure 3*c*). The mean sizes of the 95% confidence interval is 3.4 years and 13.6 years for *C1V2* and *tat*, respectively. By contrast, LS shows lower coverage probabilities but smaller confidence intervals. LS has its highest average coverage probability for *nef* (87%), but drops to 77% for *tat* (figure 3*d*). For the longest genomic region, *C1V2*, the average coverage probability is only 82%. This is likely due to the much smaller confidence interval size. The size of the 95% confidence interval is much larger for the LR method than either the LS or Bayesian methods (figure 3*c*). The LS and Bayesian methods have similar size confidence intervals, but the Bayesian method is more likely to contain the true value in the 95% confidence interval (has higher average coverage probability). The ML method has the largest RMSE and bias on average for all regions and does not provide confidence intervals.

For the Bayesian method, when the inferences are combined across all four genomic regions, the average size 95% credible set is 144 days smaller on average than with *C1V2* alone. The average probability the true integration time is in the 95% credible set is similar to the results for the longest genomic region. When the two shortest genes, *p17* and *tat*, are combined, the average size of the 95% credible set is slightly smaller than with *p17* alone (60 days), but the probability the true value is in the 95% credible set increases from 91% with *p17* alone to 95% in the combined analysis (figure 3*c*,*d*). The average RMSE is slightly smaller for the combined analysis of all genes (0.59) than with *C1V2* alone (0.67). The average RMSE is smaller when *p17* and *tat* are combined (1.39) than with *p17* alone (1.53).

### 3.2. Empirical analysis

We applied each of the four methods to HIV datasets from two studies of serial sampled HIV sequences. The first dataset comprises *nef* sequences for two patients [16]. For each patient, plasma HIV RNA was sequenced multiple times over a period of almost a decade either pre-treatment or during incompletely suppressive dual ART. After the initiation of combination ART (cART), samples from the putative reservoir were taken from at least two time points. Samples consisted of HIV RNA sequences sampled during viral blips and proviral DNA collected from whole blood and peripheral blood mononuclear cells (PBMC). The second dataset has three regions of *env* for both the patients analysed (217 and 257) and *gag* and *nef* sequences for one patient (257) [14]. For both patients, virus was sequenced from the plasma multiple times over several years prior to ART initiation. After ART initiation, viral RNA was isolated from the supernatant of QVOAs.

**Figure 4.**
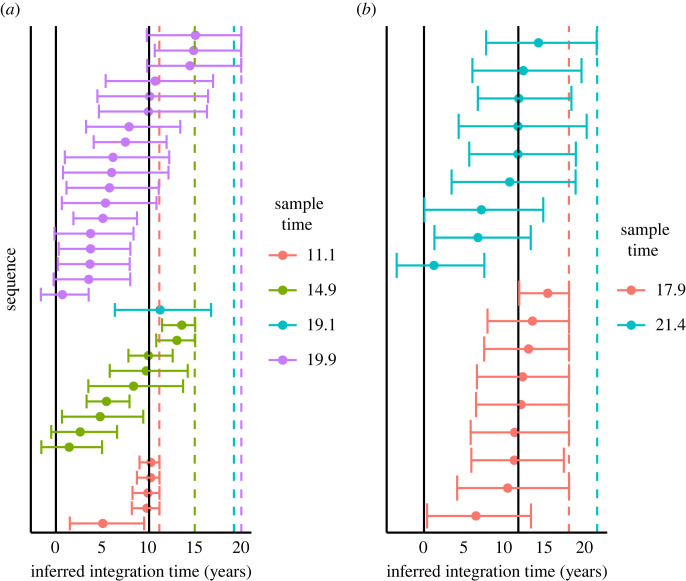
(*a*,*b*) The inferred latent integration times, in units of years after diagnosis, for patients 1 and 2, respectively, inferred using HIVtree to analyse sequence data for the *nef* gene locus. A dot indicates the posterior mean and bars represent the 95% credible interval. The solid vertical lines indicate the positive test date (left) and time of cART initiation (right) for each patient. The coloured dashed vertical lines indicate the sample times.

**Figure 5.**
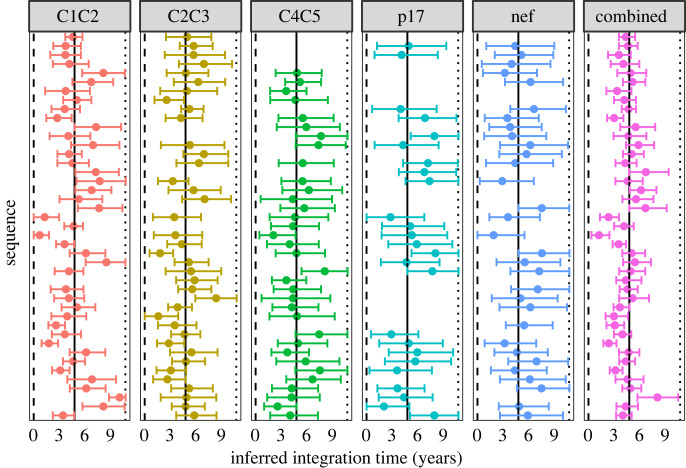
The five panels to the left each show the integration times inferred using HIVtree for a single genomic region locus. The panel to the right shows the inferred integration times when posterior distributions for the five loci are combined. A dot indicates the posterior mean and bars represent the 95% credible interval, in units of years after diagnosis. The results are from patient 257 [14]. Ten non-latent sequence were used as each available timepoint and sites with more than 75% gaps were removed from the alignment prior to analysis, as described in electronic supplementary material, section S11. The dashed line shows the infection time, the solid line shows the start of ART and the dotted line shows the sample time.

The inferred latent integration times for the patients in the first dataset obtained using HIVtree span over a decade (figure 4), similar to estimates obtained using other methods (electronic supplementary material, figure S14). However, ML and LR infer integration times that occur after the sampling time in some cases (electronic supplementary material, figure S14). For the second dataset, the point estimates, especially for the early sample times (11.1 for patient 1 and 17.9 for patient 2), tend to be concentrated near the time of ART initiation. The combined point estimates for the latency times inferred using HIVtree appear loosely clustered around the time ART began for patient 257, with narrower credible sets than the analyses on individual genomic regions (figure 5). These patterns for patient 217 are less clear, possible due to fewer genomic regions and fewer latent sequences (electronic supplementary material, figure S15). Sometimes LS gives very large confidence intervals, covering the entire area between the bounds for a sequence (electronic supplementary material, figures S16 and S19), while in other cases the confidence intervals are smaller than LR.

## 4. Discussion

Here, we have described both a phylogenetic method to infer latent integration times and a new method to simulate sequence data based on within-host viral dynamics. While there is currently only a limited amount of data suitable for this method, future research will be able to use this tool to make statistically justified conclusions about latent integration times. Our method does not directly address how the composition of the reservoir changes over time and the rates at which latent sequences enter the latent reservoir. However, accurate dating of the entry of individual sequences into the latent reservoir will likely be necessary to answer these questions.

HIVtree performs better than existing methods by a variety of metrics. The method has smaller credible/confidence intervals on average than alternative methods, while still containing the true value with high probability, resulting in more precise interval estimates of the integration dates. The RMSE of HIVtree was slightly lower on average than the other methods for the largest gene, the average RMSE is comparable across all methods, with a difference of about two months between the method with the lowest (Bayes) and highest (ML) RMSE. The larger RMSE for HIVtree for small regions is likely due to the influence of the uniformative prior in low information cases which increases bias; more informative priors based on other sources of information could help reduce RMSE.

HIVtree has several improvements over existing methods. It allows for biologically relevant bounds on latent integration times, such as requiring the latent times be older than the sample times with an option to bound the integration times at the time of infection. Among the alternative methods, only the LS method allows for such bounds. Bayesian inference also provides a sensible way to combine estimates across genes or genomic regions, while allowing for potentially different gene tree topologies due to recombination. This results in more precise estimates, especially when the sequences available are short. There is currently no alternative to the HIVtree method for jointly inferring latency times using multiple loci, nor is there a clear way to do so. Because each locus is relatively short, combining information across loci can greatly improve precision of latency time estimates. Lastly, Bayesian methods have the advantage, shared by other likelihood based methods, of well known statistical properties, such as statistical efficiency and consistency. By treating an alignment as data, HIVtree allows for full use of the available sequence data in the inference, whereas the other methods only use an inferred phylogenetic tree which may not be a sufficient statistic.

The simulation model in the study allows for the possibility of a lineage becoming latent, reactivating and potentially becoming latent again. However, the inference model assumes that latency can only occur at the tips of the tree. It is possible that some of the lineages in the trees have ancestral periods of latency that are not accounted for in the inference model. This does not appear to have a large impact on the results, as the inference method performs well on average. The simulation model did not include cases where an individual was on effective treatment and then ceased treatment. In this case, most of the virus in blood may be derived from sequences that were latent at some point in the past. For this reason, we caution against using this method for patients with a history of multiple periods of treatment with periods of uncontrolled viremia in between.

There are several avenues for improvement of HIVtree. In the current paper, to use data from multiple loci in HIVtree the marginal distributions for the latent integration times were combined. A more formal method to combine data across loci would be to jointly analyse the loci in a single model, allowing the MCMC to integrate over the node ages in each of gene trees separately while constraining the latent integration times to be the same for sequences derived from an individual infected cell. It would also be better to integrate over the different tree topologies, rather than fix the tree topology as is done in this method and existing methods. This would be most easily implemented in a program that accommodates multilocus data and estimates gene trees, such as bpp [29], rather than the parent program of HIVtree, MCMCtree.

Furthermore, despite the desirability of a diffuse prior on the node ages and latent times, the prior model in HIVtree seems to be too informative in some cases. The rank order of the nodes and the serial sampling cause the average root age of the phylogeny in the prior to be older than the user input prior. If the root age is constrained, such as by using a uniform prior, the latent times are pushed closer to the present time by the prior, which introduces a bias to the latent inferences (unpublished preliminary analysis). This means that constraining the root age to be close to the true age can increase the influence of the prior, leading to worse estimates of the latent times. Similar effects driven by constraints among node ages have been previously noted for fossil calibrations and serially sampled data [21,30]. However, the effects appear to be more pronounced when the root ages are close to the serially sampled sequences, as can result from within-host viral data. While there may be quite informative outside knowledge on the age of the root for HIV, such as the time of infection, we currently caution against forcing the root age to match the infection time when using HIVtree because this may induce bias in estimates of latent virus integration times.

The difference between the user input prior distribution on the root age and the prior observed when running the MCMC without data appears to be larger with the empirical data than with the simulated datasets. While the exact cause of this discrepancy is unknown, it may be related to the ladder-like tree topologies of the empirical data or the sampling times of the sequences. A different prior may improve some of these limitations. One option would be a serial sample coalescent prior with changing populations sizes [31,32]. This would also be more sensible to implement in a program which includes coalescent models, such as bpp. Such a prior could also allow for the incorporation of information on viral population sizes (such as from well described viral dynamic models) and knowledge of the time of infection.

The viral dynamic simulation method developed in this paper is based on well-studied models of HIV population dynamics within hosts. This is likely to be more realistic than traditional methods used to simulate phylogenies, such as constant rate birth–death processes, and it follows standard epidemiology approaches for studying viral dynamics. However, this model does not incorporate selection or recombination, which are known to be important in HIV evolution and effect tree topology. The method produces trees that are more star-like, with short internal branches, than those typically inferred in empirical studies of HIV sequences. Future work should focus on simulating selection and recombination, as well as other aspects of HIV biology, such as clonal proliferation of latently infected immune cells, which may impact tree topology and latent histories. Additionally, researchers should investigate different priors for inference that may more accurately model HIV biology and produce trees that more closely match the empirical observations, such as the ladder-like nature of many within-host HIV phylogenies.

## 5. Material and methods

Here, we provide a brief description of the materials and methods used in this paper, which are described fully in the electronic supplementary material.

### 5.1. Simulation of phylogeny

A stochastic simulation based on existing ODEs was developed to simulate tree topologies of sampled latent and active HIV sequences. In the ODE, the sizes of five populations of cells and viruses are tracked, including uninfected CD4+ target cells, productively (actively) infected CD4+ target cells, virions, replication–competent latent cells and replication-incompetent latent cells (electronic supplementary material, section S1). The start of ART prevents the infection of new cells. The stochastic model is formulated as a continuous-time Markov chain with instantaneous rates as described in the deterministic model (electronic supplementary material, section S2). The process is modelled as a jump chain. A user specified number of virions and latent cells are sampled at any number of user specified times.

A C program was written to simulate under the stochastic model. In addition to simulating population sizes, it tracks the parent–daughter relationships of all infected cells and viruses in a binary tree (electronic supplementary material, section S3). The amount of time latent in each branch is also tracked. The stochastic and deterministic models are in good agreement when population sizes are large, as expected (electronic supplementary material, figure S3). The total number of tips in the tree varied over time. The maximum number of tips in a tree was on the order of 10^8^ (electronic supplementary material, figure S3).

### 5.2. Simulation of sequence data

A separate C program was written to simulate DNA sequences given a sampled tree with branch lengths and a latent history. Sequences are simulated in the typical manner, assuming independent substitutions among sites, starting at the root of the tree and simulating forward in time towards the tips of the tree. The simulator accommodates models as general as the GTR + Γ substitution model [33,34]. No substitutions can occur while a lineage is latent. Recombination and indels were not simulated. Typically, regions with many indels are removed from alignments. Not including indel in the simulation model has the likely effect of making the sequences slightly longer and slightly more informative than if indels were simulated, but parts of the alignment were removed. The program allows an outgroup with a node age of zero to be simulated. The sequence at the root is specified by a FASTA format input file (from an existing HIV sequence, for example).

### 5.3. Sampling and simulation parameters

One-hundred trees were simulated using the stochastic simulator. Fifty viruses are sampled every year for 9 years. At year 9, ART begins. One-hundred latent cells are sampled at year 10. For each of these 100 phylogenies, 30 alignments for each of four genomic regions were generated with the DNA simulator using an outgroup. To determine the DNA substitution parameters, within-host longitudinal samples from published datasets for four regions (*tat*, *p17*, *nef*, *C1V2*) were analysed with MCMCtree (electronic supplementary material, section S6). The estimated substitution rate and length varied among the simulated regions, with *C1V2* having the highest substitution rate (*μ* = 3.56 × 10^−5^ per base per day) and the most sites (*n* = 825) and *nef* having the next highest substitution rate (*μ* = 1.34 × 10^−5^ per base per day) and number of sites (*n* = 618). *p17* has a slightly lower substitution rate than *tat* (*μ* = 8.9 × 10^−6^ per base per day versus *μ* = 9.9 × 10^−6^ per base per day), but more sites (*n* = 391 versus *n* = 132) (electronic supplementary material, table S2). *C1V2* had the lower *α* for the Γ rate variation model, meaning it has the highest variance in the substitution rate among sites. For each phylogeny and alignment, the sequences and phylogenies were then subsampled three times to generate three trees and three corresponding alignments. Specifically, 10 viruses were subsampled every year for 9 years. Twenty were subsampled at 10 years of infection. In total, 300 tree topologies were simulated, each with 20 latent and 90 non-latent randomly sampled sequences. This led to a total of 300 topologies × 30 alignments × 4 regions = 36 000 simulated datasets.

### 5.4. Maximum likelihood tree inference and rooting

To analyse the simulated datasets a rooted tree topology was first inferred for use by HIVtree and other heuristic programs. ML trees were inferred with raxml-ng using an HKY + Γ model and outgroup rooted [35,36]. Twenty-five parsimony and 25 random starting trees were used for the tree search. The outgroup was removed from the inferred tree. Both the LS and Bayesian methods use the outgroup rooted tree. For the ML method, the tree was re-rooted using root to tip regression available in the R package ape prior to analysis [22,37]. The LR method re-roots the tree using root to tip regression as part of the analysis. For LS, the sampling time was used as an upper bound for the latent lineages and the lower bound was 45 days prior to infection, while the active lineages were constrained to their sampling time. The ML and LR methods do not include additional constraints.

### 5.5. Bayesian inference

For HIVtree analyses of simulated data, an HKY + Γ model was used with 5 rate categories and the prior κ∼Γ(8,1) [35]. The prior for among site rate variation was α∼Γ(4,8). A time unit of 1000 was used with a substitution rate prior of Γ(2,200), meaning the mean was 10^−5^ per base per day. The root age prior was Γ(36.5,100). The latent times were bounded at 3.695, which is equivalent to 45 days prior to infection. Two MCMCs were run for each analysis to check for convergence. MCMC lengths and conditions for convergence are described in electronic supplementary material, section S8).

### 5.6. Combining posterior estimates from HIVtree

For combining results in Bayesian analyses of the simulated and empirical datasets, the function kdensity in the kdensity R package was used for kernel density estimation of the posterior distribution and the prior distribution of each latent time [38]. The posteriors and priors for each genomic region were multiplied according to equation (2.1), using a uniform distribution between the sample time and the upper bound for the oldest possible integration date as the desired prior. The resulting function was normalized by finding the proportionality constant using the integrate function. For the simulated datasets, the integral bounds were set to the bounds on the latent time in HIVtree, which was the sample time and 45 days prior to infection. The 0.025 and 0.975 quantiles were found using the invFunc function in the R package GoFKernel [39]. The mean for the joint posterior was found using the integrate function. For the simulated datasets, this analysis was conducted on only a third of the trees from the main simulation analysis due to the highly demanding computations involved. For a small subset of simulated data, numerical issues prevented estimation of a combined latent integration time (electronic supplementary material, section S9b).

### 5.7. Existing methods

The LR method used scripts available at: https://github.com/cfe-lab/phylodating This method uses a linear model to estimate the latent integration dates. The ML method used scripts available at: https://github.com/brj1/node.dating/releases/tag/v1.2 This method uses a pseudo-ML approach to estimate the latent integration times by fixing the mutation rate and then using ML to estimate the integration dates. The driver script provided by Jones *et al.* is available at: https://github.com/nage0178/HIVtreeAnalysis. The LS method was obtained from: https://github.com/tothuhien/lsd-0.3beta/releases/tag/v0.3.3 This method uses a LS approach to minimize the difference between the branch lengths and sample dates and infer unknown ages.

### 5.8. Empirical analysis

Datasets published from Jones *et al.* [16] and Abrahams *et al.* [14] required curation prior to analysis. Owing to the large number of sequences in the Abrahams *et al.* dataset, sequences were subsampled and alignments were edited due to gaps (electronic supplementary material, section S11). For all empirical datasets, raxml-ng was run using an HKY + Γ model [36]. Twenty-five parsimony and 25 random starting trees were used for the tree search. Trees were rooted using root to tip regression using the rtt function (implemented by Rosemary McCloskey) in the ape package available in the R package ape prior to analysis [22,37]. Each of the four methods were run on all datasets.

For the first dataset [16], HIVtree was run with a root age prior of Γ(8,60) for patient 1 and Γ(15,50) for patient 2. These priors were chosen to have an induced prior when running without data with a variance of several years and a mean several years prior to diagnosis. Latent integration times were bounded 10 years prior to diagnosis, as a very conservative oldest possible bound. In the HIVtree analysis, an HKY + Γ model was used with 5 rate categories with the prior κ∼Γ(8,1). The prior for among site rate variation was α∼Γ(4,8). A time unit of 1000 was used with a substitution rate prior of Γ(5,1000), meaning the mean was 5 × 10^−6^ per base per day. For the LS analysis, latent integration times had the same bounds of 10 years prior to diagnosis and the sample times.

For the second dataset [14], the LS and HIVtree analyses bounded the latent times at the infection times and the sample times. In the HIVtree analysis, an HKY + Γ model was used with 5 rate categories with the prior κ∼Γ(8,1). The prior for among site rate variation was α∼Γ(4,8). A time unit of 1000 was used with a substitution rate prior of Γ(2,200), meaning the mean was 10^−5^ per base per day. The root age prior was Γ(0.25,110) for all datasets. This prior was chosen to have a relatively wide variance on the root age with a mean slightly before the infection time as well as a large variance on the latent integration times. As described in the Prior Model section, the root ages are older than the given prior when run without data, and they are also different for each dataset. When running the MCMC under the prior, small changes to the prior appeared to cause little change to the posterior distribution of the latent integration times. A full description of the MCMC convergence criteria is provided in electronic supplementary material, sections 10 and 11 for the first and second datasets, respectively. The first dataset only sampled one gene, so estimates from multiple genes could not be combined. The estimates from multiple genomic regions for the second dataset were only combined for the tree with 10 non-latent sequences per sampling time and sites with gaps in over 75% of the sequences were removed from the alignment.

## Data Availability

The gene tree and the DNA simulation software packages are available at: https://github.com/nage0178/HIVtreeSimulations. The HIVtree software package is available at: https://github.com/nage0178/HIVtree. Scripts to produce the results in this paper are available at: https://github.com/nage0178/HIVtreeAnalysis. The data are provided in electronic supplementary material [40].
